# Hair and salivary cortisol and their relationship with lifestyle, mood and cognitive outcomes in premanifest Huntington’s disease

**DOI:** 10.1038/s41598-021-84726-4

**Published:** 2021-03-09

**Authors:** Travis Cruickshank, Tenielle Porter, Simon M. Laws, Mel Ziman, Danielle M. Bartlett

**Affiliations:** 1 Centre for Precision Health, Edith Cowan University, Joondalup, WA Australia; 2 Collaborative Genomics and Translation Group, School of Medical and Health Sciences, Edith Cowan University, 270 Joondalup Drive, Joondalup, WA Australia; 3 Perron Institute for Neurological and Translational Science, Nedlands, WA Australia; 4 School of Pharmacy and Biomedical Sciences, Faculty of Health Sciences, Curtin Health Innovation Research Institute, Curtin University, Bentley, WA Australia; 5 School of Medical and Health Sciences, Edith Cowan University, Joondalup, WA Australia; 6 School of Biomedical Science, University of Western Australia, Crawley, WA Australia

**Keywords:** Huntington's disease, Adrenal cortex hormones

## Abstract

Salivary cortisol dysrhythmias have been reported in some, but not all studies assessing hypothalamic–pituitary–adrenal (HPA) axis function in Huntington’s disease (HD). These differences are presumed to be due to environmental influences on temporal salivary cortisol measurement. Further exploration of HPA-axis function using a more stable and longer-term measure, such as hair cortisol, is needed to confirm earlier findings. This study aimed to evaluate hair and salivary cortisol concentrations and their associations with clinical and lifestyle outcomes in individuals with premanifest HD (n = 26) compared to healthy controls (n = 14). Participants provided saliva and hair samples and data were collected on clinical disease outcomes, mood, cognition, physical activity, cognitive reserve, sleep quality and social network size to investigate relationships between clinical and lifestyle outcomes and cortisol concentrations. Hair and salivary cortisol concentrations did not significantly differ between the premanifest HD and control groups. No significant associations were observed between hair or salivary cortisol concentrations and cognitive, mood or lifestyle outcomes. However, hair cortisol concentrations were significantly associated with disease outcomes in individuals with premanifest HD. Significant associations between hair cortisol concentrations and measures of disease burden and onset may suggest a potential disease marker and should be explored longitudinally in a larger sample of individuals with HD.

## Introduction

Hypothalamic–pituitary–adrenal (HPA) axis dysfunction has been suggested to be a key pathological feature and contributor to cognitive, mood and sleep disturbances in individuals with Huntington’s disease (HD)^1,2^. Studies in animal models and in individuals with HD have nevertheless yielded inconsistent findings, with some but not all investigations reporting acute cortisol dysrhythmias^3–5^.

Early investigations noted a significant elevation in cortisol levels in individuals with manifest HD compared to healthy age and sex matched controls^6–8^. However, more recent studies have been unable to replicate these earlier findings, consistently reporting no differences between individuals with HD and healthy controls. These inconsistent findings have been attributed to differences in sampling methods, as well as a failure to control environmental factors known to influence cortisol release.

To date most investigations have analysed cortisol release from collected blood, urine and saliva samples^6–8^. Two of the existing studies have examined the cortisol awakening response (CAR)^6,7^, which refers to the sharp increase in cortisol secretion that occurs within the first 30–45 min following awakening in the morning^9^. This biological phenomenon has been reported to display a consistent pattern in adults and exhibits good intra-individual stability across two days, particularly when total morning cortisol output is examined (area under the curve, AUC)^9–11^. While valid and well-established, these methodological approaches only provide information on short-lived stress, indicated by temporal cortisol release (< 24 h). Furthermore, while informative from a situational context, it is of utmost importance to evaluate longer-term or sustained stress, indicated by cumulative cortisol release, particularly considering the potential impact on cognitive, mood and sleep outcomes^5,8,12^, which are all negatively impacted in HD^13–15^. It is therefore crucial to identify and evaluate the sensitivity of methodological approaches aimed at examining longer-term stress and associated cumulative cortisol release.

Recent studies in healthy and neurodegenerative populations indicate that hair is a useful biological sample for the analysis of longer-term, cumulative cortisol release. In a recent investigation, Pereira et al.^16^ noted significantly higher hair cortisol concentrations in individuals with multiple sclerosis compared to healthy controls. Another investigation by Assayag et al.^17^ reported larger legion volumes and poorer cognitive performance in stroke victims with higher hair cortisol concentrations. Despite these positive findings, the utility of hair as a longer-term measure of cumulative cortisol release has not been investigated in individuals living with HD.

The primary aim of this investigation was to examine differences in hair and salivary cortisol concentrations between individuals with HD and healthy age and sex matched controls. Secondary aims of this investigation included: (1) examination of the environmental factors that significantly influence hair and salivary cortisol concentrations and (2) examination of associations between hair or salivary cortisol concentrations and cognitive and mood outcomes.

## Materials and methods

### Study design

The aim of this cross-sectional study was to compare hair and salivary cortisol concentrations between individuals with premanifest HD and healthy controls and to examine the relationships between hair or salivary cortisol concentrations and environmental, cognitive and mood measures in individuals with premanifest HD and healthy controls.

### Study approval and patient consent

The study was carried out in accordance with the Declaration of Helsinki and was approved by the North Metropolitan Area Mental Health Service (2009_16), Edith Cowan University (13145) and Monash University (CF15/117-2015000058) Human Research Ethics Committees. All participants provided written informed consent prior to undertaking study assessments.

### Participants

Twenty-six individuals with premanifest HD and 14 healthy controls participated in the study. Participants were recruited in Perth and Melbourne through the Enroll-HD study database, existing study databases, clinicians and HD community organisations (premanifest HD) and via radio and newspaper advertisement (healthy controls). Inclusion criteria for individuals with premanifest HD were as follows: (1) a cytosine-adenine-guanine (CAG) repeat length of > 39; (2) a Unified Huntington’s Disease Rating Scale (UHDRS) Diagnostic Confidence Level (DCL) of ≤ 2; and (3) a UHDRS Total Motor Score (UHDRS-TMS) of < 5 for presymptomatic or > 5 for prodromal HD^18^. Exclusion criteria for both groups were as follows: (1) concomitant neurological, cardiovascular, musculoskeletal, endocrine or metabolic disorders, (2) shift work, (3) recent or ongoing substance abuse, (4) current smoker, and (5) the inability to understand written and verbal English.

### Study procedures

Participants collected saliva samples on two consecutive mornings. Hair samples (collected by D.B) and cognitive measures (collected by T.C) were obtained from participants on one of the two saliva sampling days. Information regarding mood state and lifestyle activities was collected via self-report questionnaires during week of cortisol sampling. To maintain consistency in sampling and for pragmatic reasons, participants were asked to provide saliva samples on either two consecutive workdays or two consecutive non-workdays, but not a mix of both, with non-workdays suggested. The UHDRS-TMS and DCL assessments were performed by a qualified clinician within four weeks of collection of other study data. CAG repeat length was provided by participants and other disease characteristics, such as estimated age at onset, estimated years to onset, disease burden score and CAG-age product score (CAPs) were calculated using previously established methods^19–21^. Sociodemographic characteristics were collected via a questionnaire. Body mass index (BMI) was calculated by dividing the participant’s weight (in kg) by their height squared (m^2^), with dual energy x-ray absorptiometry (DXA; Hologic Discovery A, Waltham, MA, USA) and wall mounted stadiometer (Model 222, Seca, Hamburg, DE) used to collect weight and height, respectively.

### Cortisol measures

#### Hair cortisol

Hair samples were cut from the vertex posterior area of the head, as close to the scalp as possible. The first 3 cm of hair from the scalp end was used to assess cortisol concentrations over the previous three months, based on the assumption that hair grows at a rate of approximately 1 cm per month^22^. The hair washing and cortisol extraction procedures were carried out in line with a pre-existing protocol^23^. Briefly, hair was washed twice in isopropanol and then left to air dry. The hair was weighed and transferred to 2 mL bead beating tubes containing 2.8 mm ceramic beads (Qiagen, Germany). Hair samples were ground using the Powerlyzer 24 homogeniser and then methanol was added to the samples and incubated overnight (16 h) at room temperature for steroid extraction. Methanol was completely evaporated using a Speedvac Savant concentrator and dry samples were then eluted in 200 µL of phosphate buffered saline. Cortisol concentrations were determined using salivary cortisol enzyme-linked immunosorbent assay (ELISA) kits (IBL-International, Hamburg, Germany), according to the manufacturer’s instructions. The intra-assay coefficients of variation (CV) were below 5% and the inter-assay CV was 13.5%. The functional sensitivity of the assay was reported to be 0.138 nmol/L.

#### Salivary cortisol

Saliva samples were collected by participants in their own homes on two consecutive days. Participants were instructed on how to collect saliva samples during a face-to-face meeting with a study investigator and were provided with detailed instructions on how and when to collect saliva samples. Participants were instructed to passively drool into four separate polypropylene collection tubes (SSI Bio) at four time points in the morning at 15, 30, 45 and 60 min following awakening for morning cortisol analysis^7^. To avoid contamination of samples, participants were instructed to refrain from consuming alcohol 12 h prior and to avoid eating, drinking (with the exception of water) and brushing their teeth within the sampling time frame^7^. Participants were provided with a diary to note down their time of awakening and when saliva samples were taken and to confirm that they had not consumed anything except water and had refrained from smoking, brushing their teeth and exercising during the saliva sampling period. Participants stored saliva samples in their freezer until collection by a study investigator on the second day of sampling. Saliva samples were then stored at − 80 °C in the laboratory until analysis in duplicate using salivary cortisol ELISA kits (Salimetrics, USA) according to the manufacturer’s instructions. All intra-assay CVs were below 10% and the inter-assay CV was 7.3%. The functional sensitivity of the assay was reported to be 0.773 nmol/L. The CAR and area under the curve with respect to ground (AUC_*G*_) were calculated using the methods described by van Duijn et al.^7^. Briefly, CAR was calculated by averaging the cortisol concentrations between the 45- and 60-min time points and then subtracting the cortisol concentration of the first time point. AUC_*G*_ was calculated using the trapezoid rule to determine total morning cortisol release for each day. The variation in cortisol AUC_*G*_ values between sampling days was below 13% for both groups. Both the CAR and AUC_*G*_ values were averaged across the two sampling days^7^.

### Affective measures

The 14-item Perceived Stress Scale was used to measure stress over the previous month. The Hospital Anxiety and Depression Scale (HADS) was used to measure anxiety and depression symptomatology. The HADS is a 14-item questionnaire, with 7 items each relating to symptoms of anxiety and depression.

### Cognitive measures

The Hopkins Verbal Learning Test-Revised (HVLT-R) was used to evaluate verbal learning and memory. The One Touch Stockings (OTS) assessment was used to evaluate planning and problem solving. The Trail Making Test (TMT; Parts A and B) was used to examine attention and cognitive flexibility. The Symbol Digit Modalities Test (SDMT) was used to assess processing speed.

### Lifestyle measures

Lifestyle measures included self-report physical activity, cognitive reserve, social network, sleep quality, smoking and alcohol consumption questionnaires. Physical activity was evaluated using the Minnesota Leisure Time Physical Activity Questionnaire. Cognitive reserve was calculated using values obtained from Cognitive Activity Scale, National Adult Reading Test, occupation and education status. Smoking status (packets per week) and alcohol consumption (standard drinks per week) were evaluated using a custom questionnaire. Social network size and diversity was evaluated using the Social Network Index. Sleep quality was measured using the Pittsburgh Sleep Quality Index. These measures were purposefully selected as they have been shown to be associated with measures of disease progression in HD^24,25^ as well as cortisol regulation in the elderly and other clinical populations^26,27^.

### Statistical analysis

Based on sample size estimations, 29 participants were required per group (α = 0.05, 1 − β = 0.8)^28^. However, as this study was ancillary to another study, recruitment was limited to the participant pool of the parent study, as well as other factors such as hair length, pigment loss and hair dying.

Rstudio (RStudio Team 2015) Version 1.2.5033 for Macintosh was used for all statistical analyses. Specific statistical packages within R were used where required and if not stated are available within the base R functionality. Summary analyses of demographic, cognition and environmental variables provided means/counts and standard deviations/percentages stratified by disease classification (premanifest HD versus healthy controls. T-tests (age, perceived stress scale) and chi-squared tests (site, sex) were used to assess the differences between demographic measures.

Data normality was assessed using Shapiro–Wilk tests and analyses were undertaken accordingly. Hair cortisol and salivary cortisol data were not normally distributed. All other data fit the normal distribution. Analyses of covariance (ANCOVA), controlling for age and sex, were performed to assess differences in hair and salivary cortisol concentrations between individuals with premanifest HD and healthy controls. Associations between clinical classification, HD characteristics, cognition and environment, and cortisol levels (both hair and salivary) were investigated. Spearman’s partial correlations were used to assess relationships between variables, with age, sex and disease burden included as covariates, where appropriate.

## Results

### Demographic and clinical characteristics

Participants ranged in age from 22 to 70 (premanifest HD) and 21 to 58 (healthy controls). BMI ranged between 20.8 to 33.4 (premanifest HD) and 19.1 to 28.7 (healthy controls). Healthy controls exhibited significantly higher stress levels, as indicated by the Perceived Stress Scale, than individuals with premanifest HD (Table 1). No other differences were observed between groups across demographic and clinical characteristics.

**Table 1:** Demographic and clinical information for individuals with premanifest HD and healthy controls.

	Premanifest HD (n = 26)	Healthy controls (n = 14)	*p* value
**Sociodemographic characteristics**			
Age	43.85 (12.19)	42.21 (10.63)	0.676
Female, n (%)	21 (80.8%)	13 (92.9%)	0.578
Ethnicity			
Caucasian, n (%)	25 (96.2%)	11 (78.6%)	0.123
BMI	25.81 (3.38)	25.18 (2.98)	0.584
Medication Use			
Glucocorticoids, n (%)	0 (0%)	0 (0%)	1.000
Contraceptives, n (%)	6 (23.1%)	3 (21.4%)	0.855
**Disease characteristics**			
UHDRS-TMS	3.96 (6.89)	–	–
DCL	0.3 (0.7)	–	–
CAGn	43.15 (3.00)	–	–
Estimated age at onset	54.97 (11.91)	–	–
Estimated years to onset	5.81 (7.73)	–	–
Disease burden score	308.87 (74.11)	–	–
CAPs	0.9 (0.18)	–	–
**Neuropsychiatric characteristics**			
Perceived stress scale	16.28 (6.69)	22.79 (7.03)	0.007*
HADS (total score)	6.77 (5.62)	10.36 (6.7)	0.080

Data is reported as mean (standard deviation) unless otherwise stated. T-tests and chi-squared tests were used for comparisons between groups. Results were considered significant at *p* < 0.05* (bolded values).

*BMI* body mass index, *UHDRS-TMS* unified Huntington’s disease rating scale total motor score, *DCL* diagnostic confidence level, *CAGn* cytosine-adenine-guanine trinucleotide repeat number, *CAPs* scaled CAG-age product score, *HADS* hospital anxiety and depression scale.

### Between-group comparisons of hair and salivary cortisol concentrations

No differences were observed in cumulative hair cortisol or temporal salivary cortisol concentrations between premanifest HD and healthy control groups (Table 2).

**Table 2:** Differences in cognition and lifestyle data between individuals with premanifest HD and healthy controls.

	Premanifest HD (n = 26)	Healthy controls (n = 14)	*p* value
**Cortisol measures**			
Hair cortisol (nmol/L)	2.66 (2.06)	4.08 (4.71)	0.230
Salivary cortisol (nmol/L)			
T1 (+ 15 min post awakening)	11.36 (4.00)	11.85 (3.71)	0.619
T2 (+ 30 min post awakening)	14.27 (4.77)	13.97 (5.17)	0.803
T3 (+ 45 min post awakening)	14.12 (3.55)	14.34 (5.35)	0.815
T4 (+ 60 min post awakening)	12.68 (3.73)	13.04 (4.15)	0.838
Estimated CAR	2.04 (3.58)	1.84 (3.96)	0.977
AUC_G_ (nmol/L)	40.41 (10.86)	40.75 (13.39)	0.890
**Cognitive measures**			
Hopkins verbal learning test-revised			
Total recall	25.38 (5.06)	31.5 (2.88)	< 0.001*
Delayed recall	9.42 (1.96)	11.43 (0.94)	0.001*
Retention	93.91 (9.81)	97 (7.32)	0.309
One touch stockings			
Choices to correct	1.38 (0.42)	1.17 (0.12)	0.064
Latency to correct	12,947.35 (5554.26)	14,809.08 (8109.34)	0.396
Latency to first choice	21,183.97 (8629.6)	27,032.33 (16,006.93)	0.140
Problems solved on first choice	17.81 (4.77)	20.64 (1.86)	0.040*
Trail making test			
Part A	29.91 (13.87)	19.43 (4.83)	0.010*
Part B	67.55 (30.96)	44.64 (12.09)	0.012*
Symbol digit modalities test	53.04 (14.2)	63.64 (5.99)	0.011*
**Lifestyle measures**			
Smoking, n (%)	3 (11.5%)	0 (0.0%)	0.505
Alcohol consumption, n (%)^a^	1 (3.8%)	2 (14.3%)	0.639
Metabolic equivalents (METs)	4084.65 (7064.6)	6685.86 (8048.19)	0.297
Cognitive reserve	0.01 (2.96)	1.21 (2.5)	0.224
Number of people in social network	25.5 (25.49)	30.36 (21.82)	0.560
PSQI global score	5.8 (3.08)	5.93 (2.73)	0.897

*CAR* cortisol awakening response, *AUC*_*G*_ area under the curve with respect to ground, *PSQI* Pittsburgh Sleep Quality Index.

^a^Excessive alcohol consumption defined as greater than 14 standard alcoholic beverages per week. Data is presented as mean (standard deviation) unless otherwise stated. T-tests and chi-squared tests were used for comparisons between groups. Results were considered significant at *p* < 0.05* (bolded values).

### Between-group comparisons of cognitive and lifestyle outcomes

Individuals with premanifest HD performed significantly worse than healthy controls on measures of verbal learning and memory (HVLT-R), planning and problem solving (OTS), attention (TMT-A), cognitive flexibility (TMT-B) and processing speed (SDMT; Table 2). No other significant differences were observed for cognitive or lifestyle measures between the groups.

### Correlations between hair and salivary cortisol concentrations and cognitive and lifestyle measures

Significant associations were noted between hair cortisol concentrations and clinical disease outcomes in the premanifest HD group (Table 3). In particular, these associations were observed for disease burden score and the estimated number of years to disease onset. No other significant associations were observed between cumulative hair or temporal salivary cortisol concentrations and clinical, cognitive or lifestyle measures in the premanifest HD or healthy control groups.

**Table 3:** Spearman’s partial correlations between clinical variables and hair and salivary cortisol in individuals with premanifest HD.

Outcome	Hair cortisol		Salivary cortisol	
	*r*_*s*_-value	*p* value	*r*_*s*_-value	*p* value
**Clinical characteristics**				
CAGn	0.05	0.83	− 0.28	0.22
UHDRS-TMS	− 0.13	0.56	0.37	0.10
DBS	**0.52**	0.01*	− 0.15	0.51
CAPs	− 0.17	0.45	0.42	0.06
Estimated years to onset	**0.44**	0.04*	− 0.11	0.63
Estimated age of onset	0.19	0.39	0.19	0.41
BMI	− 0.23	0.32	0.19	0.43
HADS				
Anxiety	− 0.05	0.82	0.17	0.46
Depression	− 0.12	0.61	− 0.17	0.46
Total score	− 0.11	0.63	0.07	0.76
**Cognitive measures**				
Hopkins verbal learning test-revised				
Total recall	− 0.26	0.23	− 0.16	0.48
Delayed recall	− 0.20	0.37	− 0.31	0.17
Retention	0.04	0.86	− 0.41	0.07
One touch stockings				
Choices to correct	0.01	0.97	0.13	0.57
Latency to correct	0.15	0.50	0.26	0.25
Latency to first choice	0.00	0.99	0.33	0.12
Problems solved on first choice	0.15	0.71	− 0.06	0.82
Trail making test				
Part A	0.15	0.52	0.02	0.94
Part B	0.21	0.22	0.16	0.53
Symbol digit modalities test	− 0.16	0.44	− 0.39	0.08
**Lifestyle measures**				
Monthly METs	− 0.07	0.76	− 0.09	0.70
Cognitive reserve	− 0.07	0.76	0.40	0.08
Number of people in social network	− 0.13	0.48	− 0.11	0.58
PSQI global score	0.17	0.47	− 0.19	0.42

Data was assessed using Spearman’s partial correlations. Age, sex and disease burden score were controlled for in all analyses, except for correlations between hair and salivary cortisol and disease burden score, in which age and sex were control variables. Correlations were considered significant at *p* < 0.05 (bolded values) and *r*_*s*_-values were considered small, medium and large at *r*_*s*_ < 0.30, *r*_*s*_ > 0.30 to < 0.5 and *r*_*s*_ > 0.5, respectively.

*CAGn* cytosine-adenine-guanine trinucleotide repeat number, *UHDRS-TMS* Unified Huntington’s Disease Rating Scale Total Motor Score, *CAPs* scaled CAG-age product score, *BMI* body mass index, *HADS* Hospital Anxiety and Depression Scale, *METs* metabolic equivalents, *PSQI* Pittsburgh Sleep Quality Index.

## Discussion

Abnormalities in cortisol release have been reported previously in individuals with HD and have been suggested to be an indicator of hypothalamic–pituitary–adrenal (HPA) axis pathology^29,30^. However, these studies used salivary cortisol as the principal output, which is prone to temporal changes in response to environmental and psychological stimuli. Accumulative cortisol in hair has been proposed as a measure of longer-term or sustained stress. Here, we examined, for the first time, hair cortisol concentrations in individuals with premanifest HD in comparison to healthy controls. Furthermore, we assessed the relationships between cumulative cortisol in hair and shorter-term temporal salivary cortisol and clinical, cognitive and lifestyle measures in individuals with premanifest HD. We report no significant differences between groups in longer-term or sustained stress, as indicated by cumulative hair cortisol concentrations. Furthermore, we note no significant associations between hair or salivary cortisol concentrations and environmental and clinical outcomes.

Previous studies have noted significant differences in saliva cortisol concentrations between individuals with HD and healthy controls^7,8^. We were unable to replicate these earlier findings in our cohort. In particular, we found no differences in hair and salivary cortisol concentrations between individuals with premanifest HD and healthy controls. This was unexpected. Given that regulation of the CAR, and cortisol release in general, involves the hippocampus, amygdala, prefrontal cortex and suprachiasmatic nucleus^31,32^ and considering the known pathological changes to these structures to varying extents in HD^29,30,33,34^, it was anticipated that differences between hair and salivary cortisol metrics would be observed. The reason for the discrepancy in findings is not known, however it is noteworthy that, contrary to previous reports^7,35^, stress levels were significantly lower in the premanifest HD group than in healthy controls. Interestingly, cumulative hair cortisol concentrations were found to be associated with disease burden score and the estimated number of years to disease onset, initially suggesting that it may be a potential candidate for a biological marker of disease progression. However, considering no differences were observed in cumulative hair cortisol concentrations between the groups, this may represent a coincidental finding. In addition, CAR metrics did not appear to be a sensitive marker in this group, despite earlier findings^7^. Nevertheless, both cortisol measurements should be evaluated longitudinally to definitively assess whether they change over time as the disease progresses.

Associations between clinical outcomes, including mood and cognitive outcomes, and temporal salivary cortisol release have been reported previously in HD^5,8^. Shirbin et al. reported associations between short-term salivary cortisol release and depressive symptomology and performance on verbal learning and memory tasks^5,8^. These earlier findings were not reflected in the present study. In particular, no associations were observed between hair or salivary cortisol release and cognitive and mood outcomes in individuals with HD. It is possible that these relationships do not arise until late in the disease course, where symptoms of the disease progressively emerge.

Preclinical studies have previously documented positive effects of environmental enrichment on HPA-axis structures, including the adrenal glands, which are involved in the regulation of glucocorticoids^36^. Studies in older adults and other clinical populations, including schizophrenia, have additionally reported a negative effect of unhealthy lifestyle behaviours, such as physical inactivity and social isolation on cortisol regulation^27,37^. It was therefore hypothesised that lifestyle factors, particularly physical activity, cognitive reserve and social network size would be negatively associated with cortisol levels such that greater physical activity, cognitive reserve and social network size would be associated with lower cortisol levels. Our findings did not support this hypothesis, with no associations observed between hair or salivary cortisol levels and any of the lifestyle factors. However, it should be noted that this study adopted a cross-sectional design, it is possible that associations between cortisol and lifestyle outcomes may arise over time, particularly as the disease advances.

Several limitations need to be considered when interpreting our findings. First, this was a cross-sectional investigation of associations between cortisol concentrations and clinical and environmental outcomes, subsequently causation cannot be determined. Second, this study had a relatively small sample of participants with premanifest HD and healthy age and sex matched controls. Therefore, it is not known whether associations between cortisol and clinical and environmental outcomes manifest later in the disease course, which has been described, albeit only in a few studies previously. It is important to note that this study consisted predominantly of female participants. While this was not intended, this may bias the results, particularly as females may have different cortisol rhythms to males and these are influenced by menstrual events. It is also noteworthy that the timing of saliva sampling was self-reported, which can be influenced by responder and recall bias. This bias might be overcome in the future by using tubes with timing caps, which has been suggested by Stalder et al.^38^. Finally, cortisol has been shown to be influenced by seasonal changes. Participants in this study were sampled in Australia only (Melbourne and Perth) during spring, when there is progressively greater sunlight and better weather conditions. This may be in contrast to previous studies that may have taken cortisol samples in winter months, when there is less sunlight and poorer weather conditions. Future studies are needed to evaluate changes in hair and salivary cortisol, and the potential associations with clinical and environmental outcomes, longitudinally.

Despite the abovementioned limitations, it is important to note that this is the first study to investigate longer-term or sustained stress as indicated by cumulative cortisol concentrations in hair in individuals with premanifest HD. Furthermore, this is the first study to evaluate associations between longer-term or sustained stress, indicated by cumulative cortisol release, and shorter-lived stress, indicated by temporal cortisol release, and environmental outcomes in individuals with premanifest HD. In contrast to previous findings, we observed no differences in hair or salivary cortisol concentrations between individuals with premanifest HD or healthy controls. In addition, we found no associations between cortisol concentrations and mood, cognitive and environmental outcomes. Small to moderate associations were found between hair cortisol concentrations and measures of disease burden, however, as no differences were observed between groups this is likely a spurious finding. Nevertheless, this latter finding provides impetus for further investigation of longer-term or sustained cortisol and associations with clinical and environmental outcomes longitudinally.

## Data Availability

The datasets generated during and/or analysed during the current study are available from the corresponding author on reasonable request.
